# Dopamine D1- and D2-like receptors oppositely regulate lifespan via a dietary restriction mechanism in *Caenorhabditis elegans*

**DOI:** 10.1186/s12915-022-01272-9

**Published:** 2022-03-23

**Authors:** Yizhou Jiang, Uma Gaur, Zhibai Cao, Sheng-Tao Hou, Wenhua Zheng

**Affiliations:** 1 Centre of Reproduction, Development & Aging and Institute of Translation Medicine, Faculty of Health Sciences, University of Macau, Avenida de Universidade, Taipa, Macau, China; 2 Brain Research Centre and Department of Biology, Southern University of Science and Technology, 1088 Xueyuan Blvd, Nanshan District, Shenzhen, Guangdong Province China

**Keywords:** Dopamine, Lifespan, *Caenorhabditis elegans*, Aripiprazole, Dietary restriction

## Abstract

**Background:**

Despite recent progress in understanding the molecular mechanisms regulating aging and lifespan, and the pathways involved being conserved in different species, a full understanding of the aging process has not been reached. In particular, increasing evidence suggests an active role for the nervous system in lifespan regulation, with sensory neurons, as well as serotonin and GABA signaling, having been shown to regulate lifespan in *Caenorhabditis elegans* (*C. elegans*). However, the contribution of additional neural factors, and a broad understanding of the role of the nervous system in regulating aging remains to be established. Here, we examine the impact of the dopamine system in regulating aging in *C. elegans*.

**Results:**

We report that mutations of DOP-4, a dopamine D1-like receptor (D1R), and DOP-2, a dopamine D2-like receptor (D2R) oppositely affected lifespan, fast body movement span, reproductive lifespan, and developmental rate in *C. elegans*. Activation of D2R using aripiprazole, an antipsychotic drug, robustly extended both lifespan and healthspan. Conversely, inhibition of D2R using quetiapine shortened worm lifespan, further supporting the role of dopamine receptors in lifespan regulation. Mechanistically, D2R signaling regulates lifespan through a dietary restriction mechanism mediated by the AAK-2-DAF-16 pathway. The DAG-PKC/PKD pathway links signaling between dopamine receptors and the downstream AAK-2-DAF-16 pathway to transmit longevity signals.

**Conclusions:**

These data demonstrated a novel role of dopamine receptors in lifespan and dietary restriction regulation. The clinically approved antipsychotic aripiprazole holds potential as a novel anti-aging drug.

**Supplementary Information:**

The online version contains supplementary material available at 10.1186/s12915-022-01272-9.

## Background

The biological mechanisms of aging are still not well-understood, despite several conserved aging-regulatory pathways that have been identified from yeasts to humans [1]. Increasing evidence suggests that the nervous system plays an active role in the aging process. For example, studies have shown that sensory neurons play an essential role in lifespan regulation [2, 3]. Serotonin signal was reported to antagonistically modulate longevity through different serotonin receptors [4]. Recently, inhibitory neurons gamma-aminobutyric acid (GABA) signaling has also been found to regulate lifespan in *C. elegans* [5]. However, much remains to be learned concerning the role of the nervous system in the regulation of the aging process.

Whether the dopamine system regulates the aging process and lifespan is unclear. Dopamine is a biogenic amine neurotransmitter, which primarily modulates behavioral outputs in response to environmental conditions [6, 7]. For example, the dopamine system functions in behaviors like reward-seeking and physical mobility and is known to be vulnerable to the effects of aging. Once released from presynaptic terminals, dopamine activates two classes of G protein-coupled receptors: D1 and D2 classes of dopamine receptors [8]. In *C. elegans*: DOP-1 and DOP-4 belong to D1-like dopamine receptors (D1R), while DOP-2 and DOP-3 belong to D2-like dopamine receptors (D2R) [9–11]. A previous study showed that worms bearing a mutation in a dopamine biosynthesis gene *cat-2* had a normal lifespan, suggesting that solely reducing dopamine production does not affect longevity [4]. However, because D1R and D2R oppositely regulate a series of behaviors in *C. elegans*, including decision-making, basal slowing, and food response [12–14], it is important to determine the specific roles of each of the dopamine receptors in regulating the lifespan of *C. elegans*.

Because of its short lifespan, observable age-related phenotypes, and conserved aging-related biological pathways [15, 16], *C. elegans* is one of the most widely used model organisms to study aging. Despite its simple nervous system, *C. elegans* possesses a conserved dopamine system to that of the mammalian nervous system, including biosynthetic enzymes responsible for dopamine synthesis, mechanisms for synaptic release, and the expression of dopamine receptors [17]. These features made *C. elegans* a suitable genetic model system for investigating the role of the dopamine system in longevity.

In the present study, we demonstrate that dopamine D1- and D2-like receptors oppositely regulate worm lifespan through a dietary restriction (DR) mechanism. Using molecular genetics approach and pharmacological tools, we teased out how dopamine receptors send longevity signals through their G-protein-coupled signaling transduction pathways to downstream DR-related pathway. Our findings uncover a novel mechanism of dopamine signaling in DR and lifespan regulation. Notably, aripiprazole, a clinically widely used antipsychotic drug, robustly extend both the lifespan (> 50%) and healthspan (> 80%) of *C. elegans* by activating the dopamine receptor-mediated pathway. The surprise finding of aripiprazole holds promise for further development as a potentially safe, novel anti-aging drug.

## Results

### Dopamine D1- and D2-like receptors have opposite effects on the lifespan of *C. elegans*

**Fig. 1:**
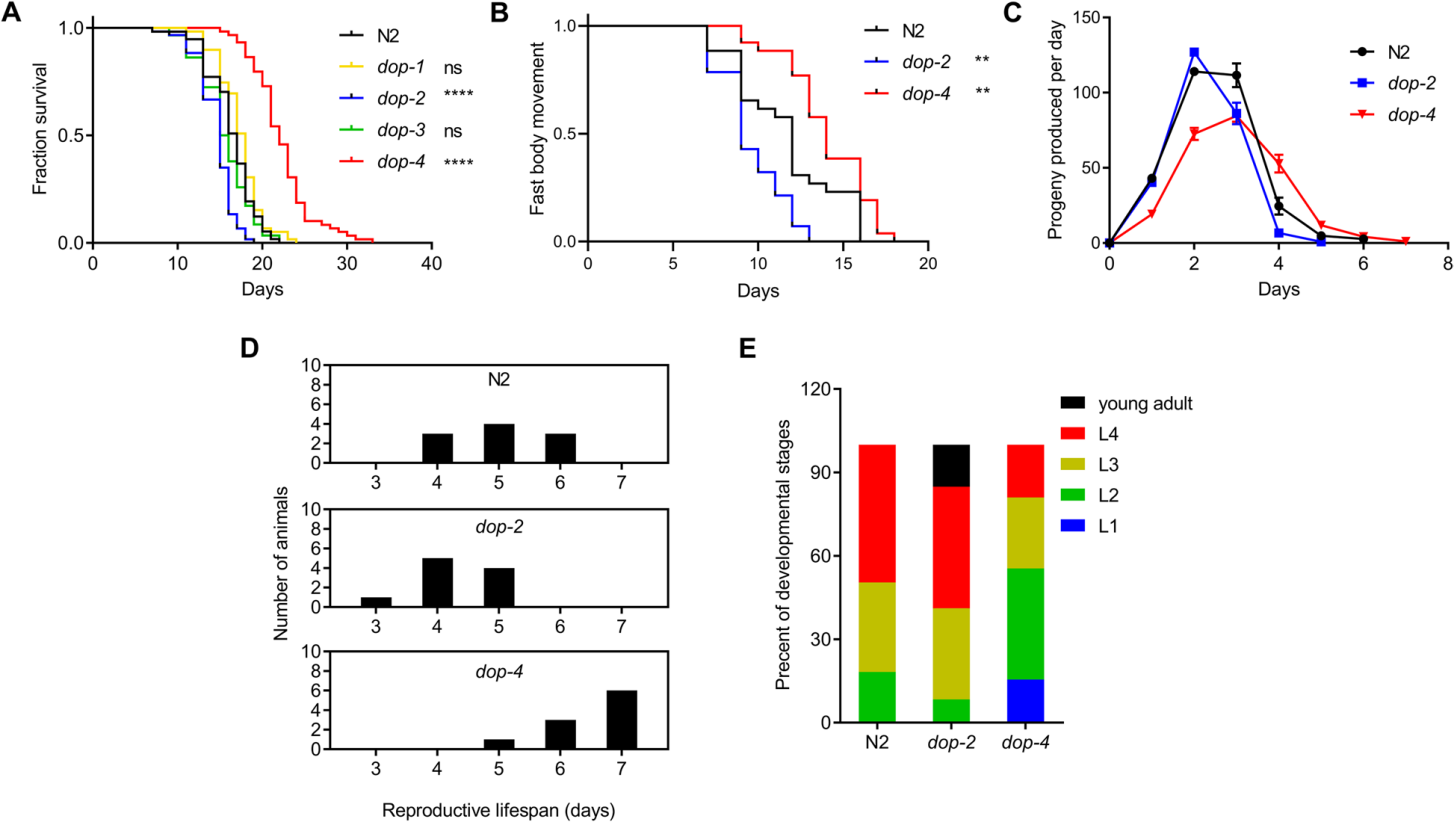
Dopamine D1- and D2-like receptors oppositely regulate lifespan in *C. elegans*. **A** Survival curves of different worm strains cultured at 20 °C (mutant strains were compared with the wild-type N2: ns = not significant; **** indicated *P* < 0.0001, log-rank test). **B** Fast body movement spans of different worm strains cultured at 20 °C (mutant strains were compared with the N2: **, *P* < 0.01, log-rank test). **C** Progeny produced per day by different worm strains. **D** The number of worms with different reproductive lifespans. **E** The developmental stage of different worm strains reached 2.5 days post-hatching. (see Additional file 2 for supporting data). Experiments were performed in three independent biological replicates

To determine the role of each dopamine receptor in worm lifespan, worm strains carrying mutations on each of the four dopamine receptor genes, including *dop-1, dop-2, dop-3*, and *dop-4*, were used for lifespan assay. Intriguingly, the *dop-2* mutant showed a significantly reduced lifespan (− 11.8%, *P* < 0.0001) than N2 animals, whereas the *dop-4* mutant was significantly long-lived (+ 29.4%, *P* < 0.0001) compared with N2 animals. The *dop-1* and *dop-3* mutants also showed changes in lifespan (at + 5.9% and − 8.8%, respectively) compared with N2 animals, which were not statistically significant (*P* = 0.0765, *P* = 0.2468, respectively, Fig. 1A). These data demonstrated that signaling through D2R (DOP-2) extends lifespan while signal through D1R (DOP-4) shortens it. The lack of statistical significance in *dop-1* and *dop-3* mutants-induced changes in lifespan indicated that DOP-4 and DOP-2 are the major subtypes of D1R and D2R in regulating lifespan, respectively.

Lifespan extension has been associated with slowed locomotory and reproductive aging, as well as delayed development [18–20]. To further understand the roles of dopamine receptors in the aging process, we next examined the fast body movement span, reproductive lifespan, and developmental rate of these mutants. Compared to N2 animals, the *dop-2* mutant showed a shorter reproductive lifespan and fast body movement span than N2 animals, whereas the *dop-4* mutant had an extended reproductive lifespan and fast body movement span (Fig. 1B–D). The *dop-2* mutant developed faster, whereas the *dop-4* mutant showed a slower developmental rate (Fig. 1D). These data further supported the idea that specific dopamine receptors played a selective and unique role in lifespan regulation.

### Pharmacological activation or inhibition of D2R exerts opposite effects on the lifespan of *C. elegans*

**Fig. 2:**
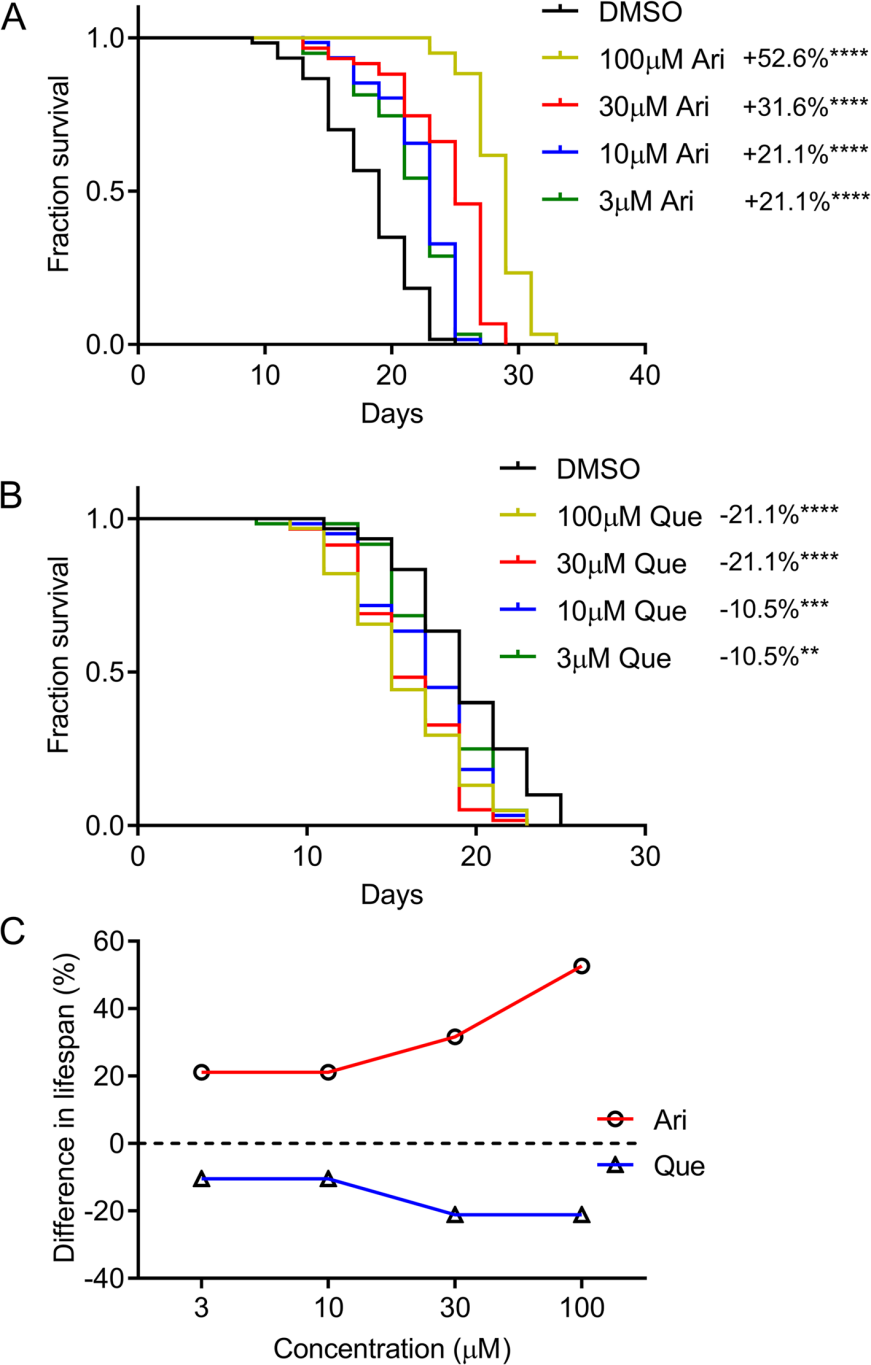
Pharmacological activation or inhibition of D2R exerts opposite effects on the lifespan of *C. elegans*. **A** Survival curves of wild-type (N2) worms cultured at 20 °C on NGM plates containing different concentrations of aripiprazole (Ari). DMSO treatment was used as a control. **B** Survival curves of N2 worms treated with the indicated concentrations of quetiapine (Que) or DMSO at 20 °C. (aripiprazole and quetiapine treatment were compared with the DMSO treatment: **indicated *P* < 0.01. ***, *P* < 0.001. ****, *P* < 0.0001, log-rank test). **C** Dose-response curve of aripiprazole (Ari) and quetiapine (Que) treatment. (see Additional file 2 for supporting data). Experiments were performed in three independent biological replicates

To further confirm the role of D2R in extending lifespan in *C. elegans*, a pharmacological approach employing aripiprazole, a D2R agonist, and quetiapine, a D2R antagonist, were used to determine the effect on worm lifespan [21, 22]. Wild-type N2 worms were treated with aripiprazole and quetiapine at concentrations ranging from 3 to 100 μM. Robust lifespan extensions were observed in aripiprazole-treated worms. Aripiprazole at 3 μM concentration significantly extended the median lifespan of N2 worms by 21.1% (*P* < 0.0001). Worms exposed to 100 μM of aripiprazole reached a maximum lifespan extension of up to 52.6% (*P* < 0.0001) (Fig. 2A). In contrast, quetiapine treatment dose-dependently shortened worm lifespan (Fig. 2B). Dose-response curves are shown in Fig. 2C. These findings further support a key role of D2R in regulating worm lifespan.

### Aripiprazole extends the lifespan of C. elegans through DOP-2

**Fig. 3:**
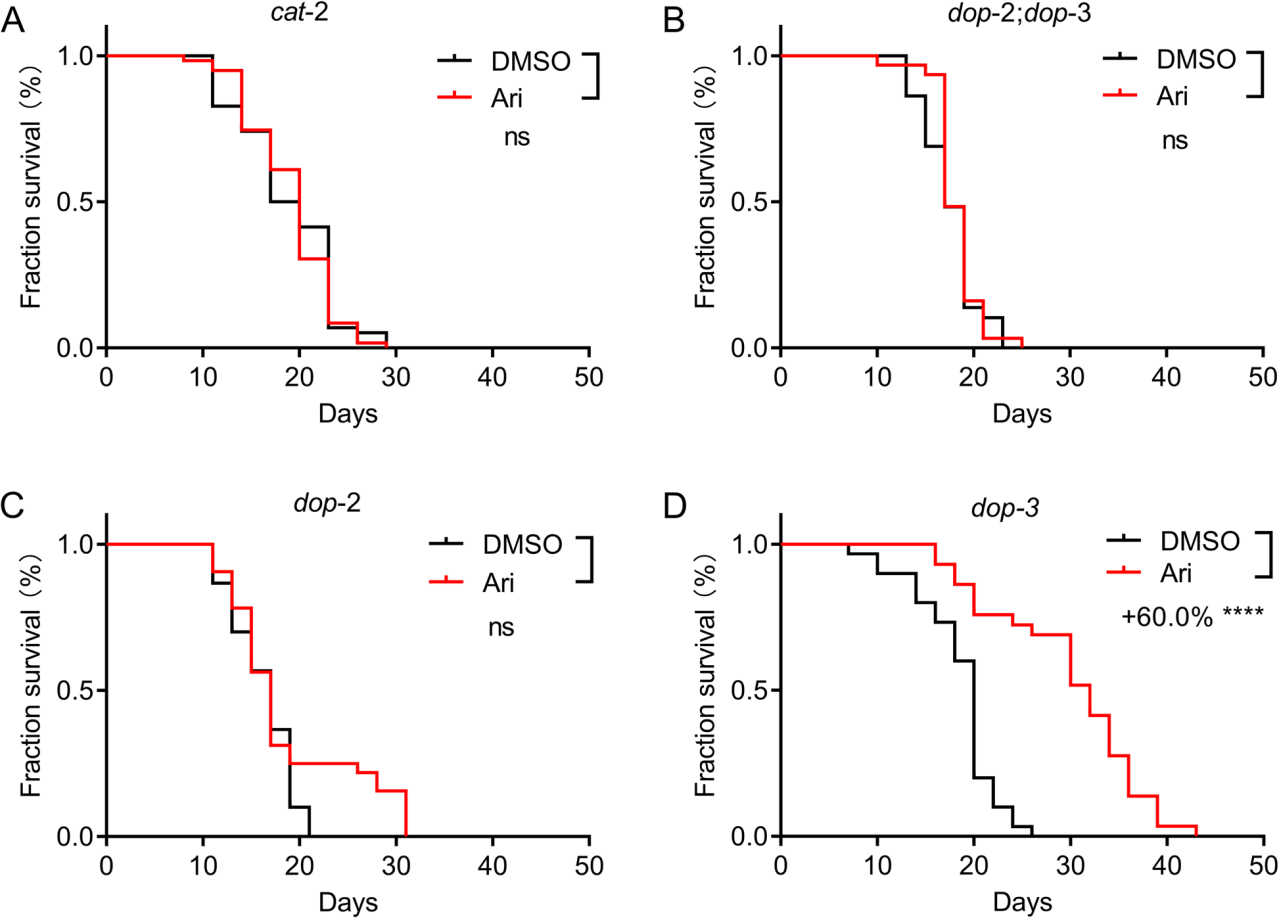
Aripiprazole extends the lifespan of *C. elegans* through DOP-2. Survival curves of mutant *cat-2* (**A**); *dop-2; dop-3* (**B**); *dop-2* (**C**); *dop-3* (**D**) treated with 100 μM of aripiprazole or DMSO at 20 °C (comparison between DMSO and aripiprazole (Ari) treatment: ns = not significant with *P* > 0.05; ****indicated *P* < 0.0001, log-rank test) (see Additional file 2 for supporting data). Experiments were performed in three independent biological replicates

To determine whether aripiprazole-mediated lifespan extension is dependent on D2R, we first tested the drug on dopamine synthesis-deficient mutant *cat-2*. Aripiprazole failed to extend lifespan in the *cat-2* mutant (Fig. 3A), suggesting that the lifespan extension effect of aripiprazole requires dopamine signaling. We next employed a *dop-2; dop-3* double mutant and found that it was insensitive to aripiprazole treatment (Fig. 3B). To investigate which of these two receptors contributes to the lifespan extension effect, we tested every single mutant. The results showed that aripiprazole did not extend the lifespan of *dop-2* mutant (Fig. 3C), while *dop-3* mutant showed a robust lifespan extension upon aripiprazole treatment (Fig. 3D). These findings demonstrated that lifespan extension by aripiprazole was conferred by its action on DOP-2.

### Aripiprazole mediates lifespan extension through D2R signaling

**Fig. 4:**
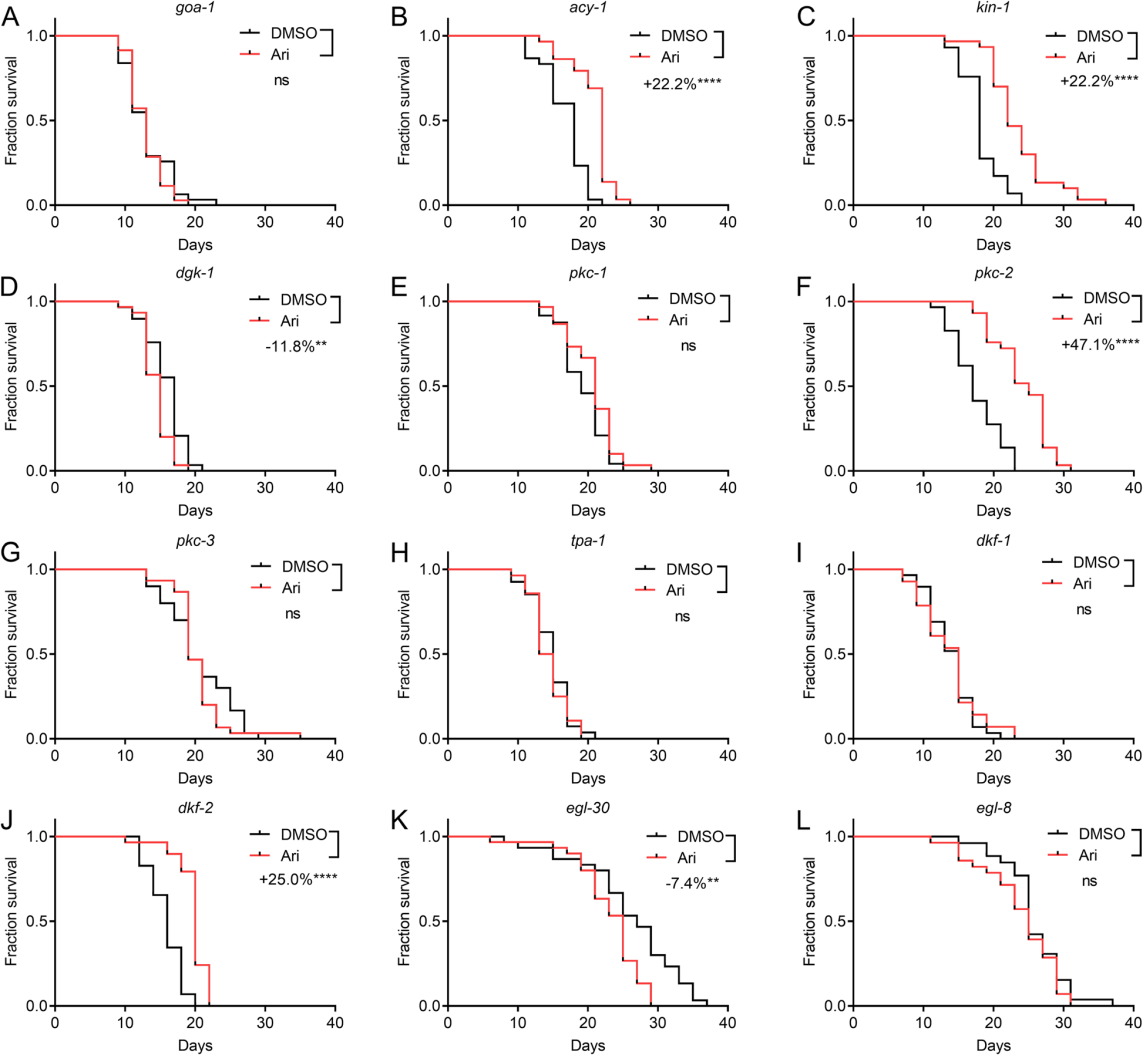
Aripiprazole mediates lifespan extension through D2R signaling. Survival curves of mutant *goa-1* (**A**), *acy-1* (**B**), *kin-1* (**C**), *dgk-1* (**D**), *pkc-1* (**E**), *pkc-2* (**F**), *pkc-3* (**G**), *tpa-1* **(H**), *dkf-1* (**I**), *dkf-2* (**J**), *egl-30* (**K**), and *egl-8* (**L**) treated with 100 μM of aripiprazole (Ari) or DMSO at 20 °C (comparison between DMSO and Ari treatment: ns = not significant with *P* > 0.05; **indicated *P* < 0.01. ****, *P* < 0.0001 with log-rank test) (see Additional file 2 for supporting data). Experiments were performed in three independent biological replicates

DOP-2 signals through Gαo pathways [8, 23]. To identify DOP-2 downstream effectors mediating the aripiprazole-induced pro-longevity effect, we first tested a mutant lacking GOA-1, the *C. elegans* ortholog of Gαo protein [24]. As expected, GOA-1 is required for aripiprazole-induced extension of lifespan (Fig. 4A). D2R inhibits adenylyl cyclase and thus suppresses the production of intracellular cyclic AMP (cAMP) and the activity of protein kinase A (PKA) [25]. The cAMP-PKA pathway has been shown to mediate lifespan and DR responses [26–28]. *acy-1* encodes adenylyl cyclase and regulates cAMP production in *C. elegans* [29]. The *C. elegans* genome encodes a PKA catalytic subunit (KIN-1) and a PKA regulatory subunit (KIN-2) [30–32]. The binding of cAMP to KIN-2 results in the release of active KIN-1 [29]. The results showed that the lifespan extension by aripiprazole treatment was only partly dependent on ACY-1 and KIN-1 (Fig. 4B, C).

Another important downstream factor of GOA-1 is DGK-1, the *C. elegans* ortholog of diacylglycerol kinase [33]. The GOA-1/DGK-1 pathway inhibits the production of diacylglycerol (DAG), thus antagonizes the effect of the EGL-30/EGL-8 pathway [34, 35]. DGK-1 was indeed essential for aripiprazole-induced lifespan extension (Fig. 4D). DAG could activate downstream molecules, including protein kinase C (PKC) and protein kinase D (PKD) [36, 37]. The worm genome encodes four PKC homologs (*tpa-1, pkc-1, pkc-2* and *pkc-3*) and two PKD homologs (*dkf-1* and *dkf-2*) [5]. Among the four PKC homologs, *pkc-1, pkc-3* and *tpa-1* were required for aripiprazole to extend lifespan, whereas *pkc-2* were dispensable (Fig. 4E–H). For the two PKD homologs, aripiprazole-mediated lifespan extension was dependent on *dkf-1* rather than *dkf-2* (Fig. 4I, J). These findings suggested that aripiprazole may extend lifespan through both PKC and PKD. To further confirm the role of the DGK-PKC/PKD pathway, we also tested *egl-30* and *egl-8* mutants, both of which are supposed to have impaired DAG production and thus reduced PKC/PKD activity [34]. As expected, aripiprazole failed to extend lifespan in both mutants (Fig. 4K, L), further supported the key role of the DGK-PKC/PKD pathway in aripiprazole-mediated lifespan extension. Taken together, aripiprazole mediates lifespan extension through GOA-1-DGK-1-PKC/PKD, but may only be partly dependent on the cAMP-PKA pathway.

### Aripiprazole extends worm lifespan through a Dietary Restriction (DR) mechanism

**Fig. 5:**
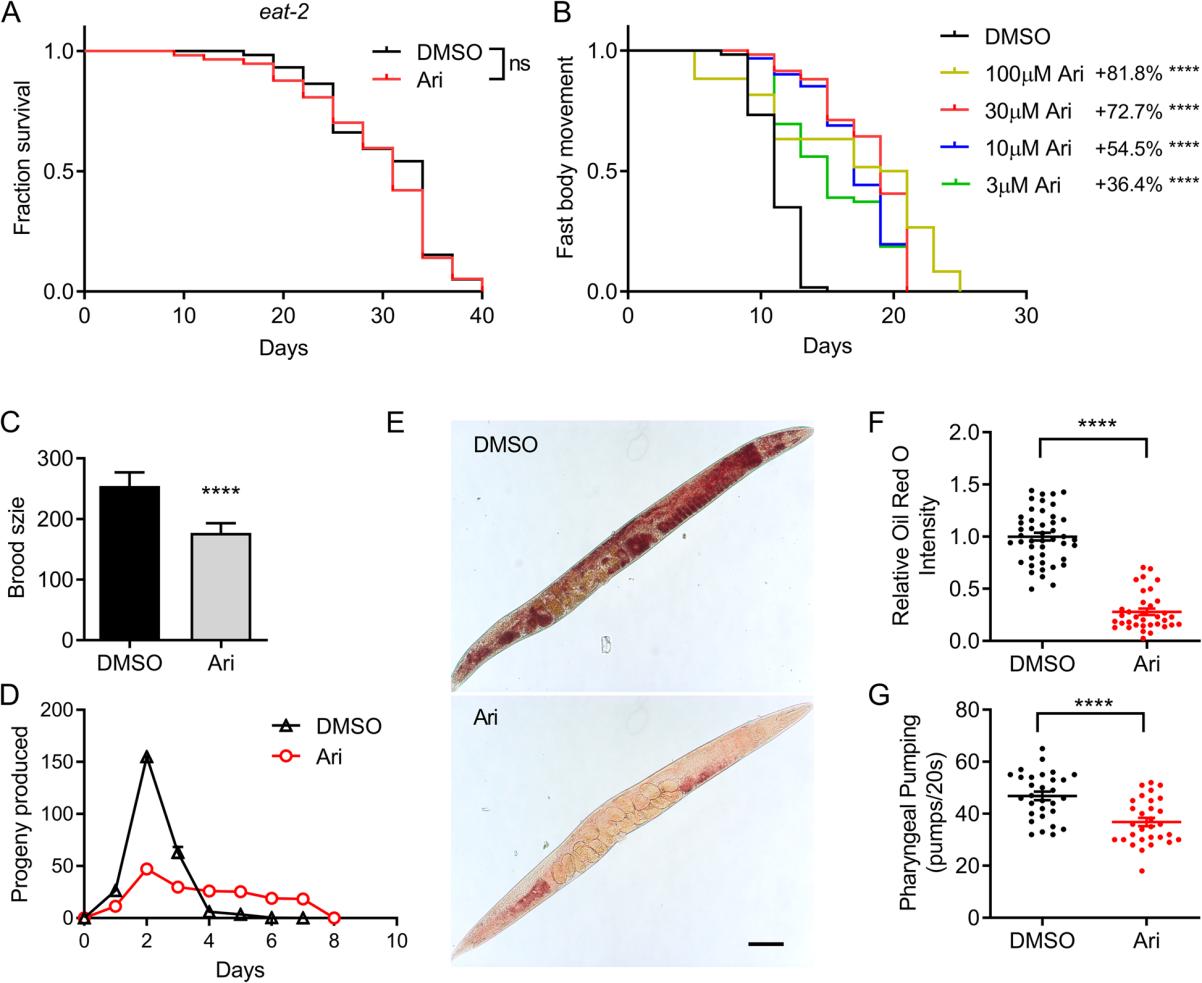
Aripiprazole extends worm lifespan through a dietary restriction mechanism. **A** Survival curves of the *eat-2* mutant treated with 100 μM of aripiprazole (Ari) or DMSO at 20 °C (comparison between DMSO and Ari treatment: ns = not significant, *P* > 0 .05. **, *P* < 0.01. ****, *P* < 0.0001, log-rank test). **B** Fast body movement spans of wild-type (N2) worms cultured at 20 °C on NGM plates containing indicated concentrations of aripiprazole (Ari) or DMSO. **C**, **D** Changes in brood size (**C**) and reproductive span (**D**) of N2 animals treated with or without 100 μM of aripiprazole (Ari) (comparison between DMSO and aripiprazole (Ari) treatment: ****, P < 0.0001, *t*-test). **E** Representative images of ORO staining of worms treated with or without aripiprazole (Ari) for 7 days. Scale bar = 200 μm. **F** Relative ORO intensity of worms treated with or without aripiprazole for 7 days (comparison between DMSO and aripiprazole (Ari) treatment: ****, *P* < 0.0001, *t*-test). **G** Pharyngeal pumping rate of N2 worms treated with 100 μM of aripiprazole (Ari) or DMSO for 5 days (comparison between DMSO and Ari treatment: **** indicated *P* < 0.0001, *t*-test). (see Additional file 2 for supporting data). The brood size assay was performed in two independent biological replicates. Other experiments were performed in three independent biological replicates

DR robustly delays the aging process in many species [38]. Several DR-related phenotypes in *C. elegans* included increased healthspan, decreased feeding behavior, reduced brood size, prolonged reproduction period, and reduced lipid storage [39, 40]. Therefore, we wanted to know whether dopamine signaling and aripiprazole treatment can modulate lifespan through DR mechanisms. We first tested aripiprazole on a long-lived *eat-2* mutant, a genetic model of DR with a deficit in pharyngeal pumping [41]. Aripiprazole did not further extend the lifespan of the *eat-2* mutant (Fig. 5A), indicating that the lifespan benefits of aripiprazole were indeed conferred by a DR mechanism. Several DR-related phenotypes were also examined. Besides the extended lifespan, the healthspan reflecting the quality of the extended lifespan was also of great significance for healthy aging. Aripiprazole increased the healthspan of *C. elegans* in a dose-dependent manner which was coupled with its effect on lifespan. Aripiprazole at 100 μM resulted in a maximum increment (87.5%) on the healthspan of N2 animals (Fig. 5B). Aripiprazole treatment also reduced the total progeny produced per worm, even with a more extended reproduction period (Fig. 5C, D). Furthermore, oil red O staining showed that aripiprazole-treated worms had reduced lipid accumulation (Fig. 5E, F). Pharyngeal pumping rate positively correlates with food intake in *C. elegans*. One direct cause of the DR-like effect is reduced food intake resulted from decreased pharyngeal pumping rate, like that observed in the *eat-2* mutant. As shown in Fig. 5G, aripiprazole treated N2 worms displayed a reduced pharyngeal pumping rate, further suggested that aripiprazole triggered a DR-like state in *C. elegans*.

### Aripiprazole mediates DR-like lifespan extension through the AAK-2-DAF-16 pathway

**Fig. 6:**
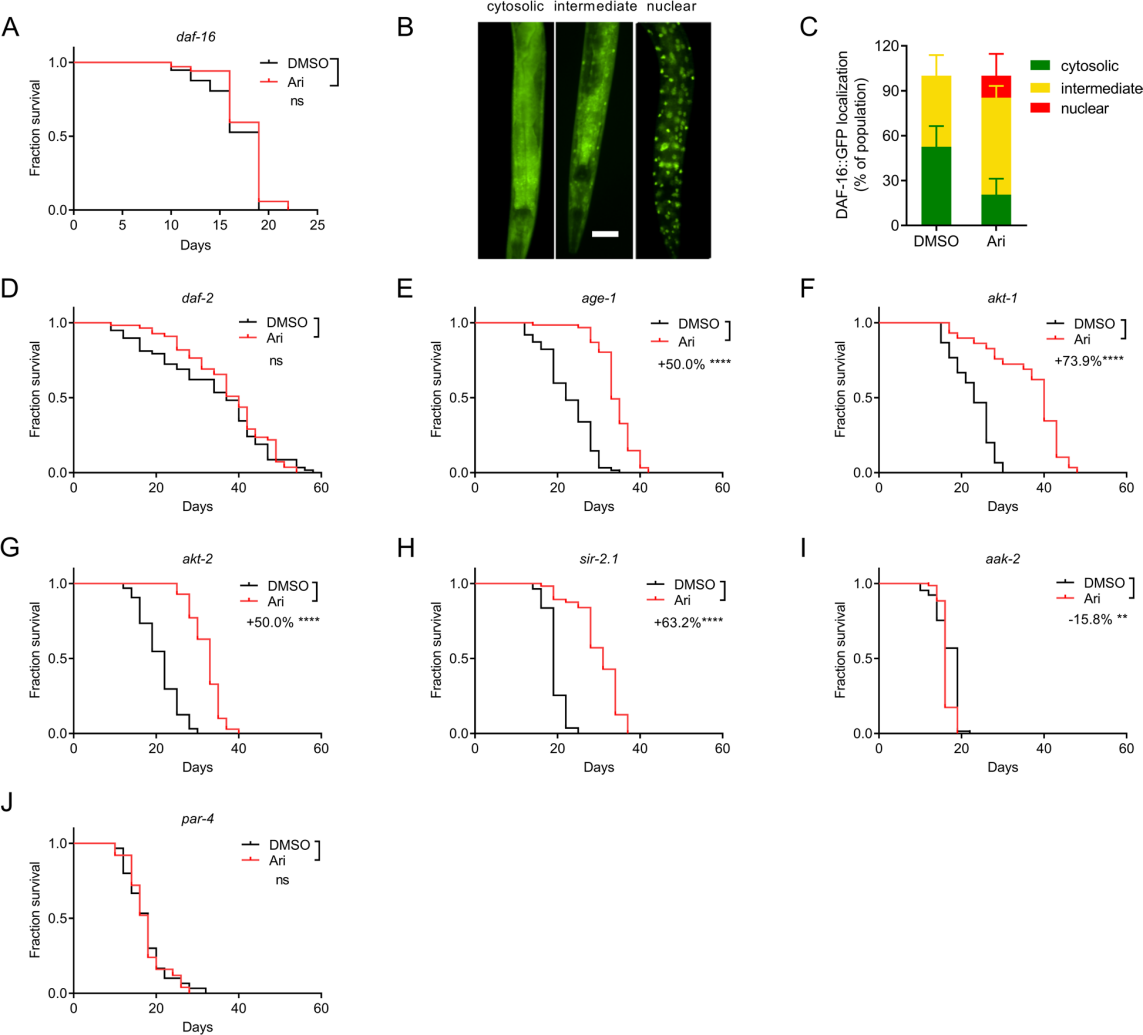
Aripiprazole mediates DR-like lifespan extension through the AAK-2-DAF-16 pathway. **A** Survival curves of the *daf-16* mutant treated with 100 μM of aripiprazole (Ari) or DMSO at 20 °C (comparison between DMSO and aripiprazole (Ari) treatment: ns = not significant with *P* > 0.05, log-rank test). **B** The representative images of worms having cytosolic, intermediate, and nuclear DAF-16 localization in TJ356 transgenic strains. Scale bar = 50 μm. **C** The percentile of TJ356 animals treated with 100 μM aripiprazole (Ari) or DMSO displaying cytosolic, intermediate, or nuclear localization. **D**–**J** Survival curves of mutant *daf-2* (**D**), *age-1* (**E**), *akt-1* (**F**), *akt-2* (**G**), *sir-2.1* (**H**), *aak-2* (**I**), *par-4* (**J**) treated with 100 μM of aripiprazole (Ari) or DMSO at 20 °C (comparison between DMSO and aripiprazole (Ari) treatment: ns = not significant with *P* > 0.05. ** indicated *p* < 0.01. ****, *p* < 0.0001, log-rank test) (see Additional file 2 for supporting data). Experiments were performed in three independent biological replicates

To further dissect the possible mechanisms of aripiprazole-mediated DR-like lifespan extension, we examined DAF-16, a *C. elegans* homolog of mammalian FOXO transcription factor known to play a central role in lifespan and DR regulation [42]. Remarkably, the lifespan extension effect of aripiprazole was abolished entirely in the *daf-16* mutant (Fig. 6A). DAF-16 locates in the cytosol under normal conditions. Once activated, DAF-16 becomes translocated to the nucleus to trigger the transcription of various genes that regulate stress resistance, metabolism, reproduction, and longevity. Indeed, TJ356 worms with green fluorescent protein (GFP)-tagged DAF-16 showed an increased accumulation of DAF-16 in the nucleus once treated with aripiprazole (Fig. 6B, C), suggesting that aripiprazole mediates DR-like lifespan extension through DAF-16.

In DR-like mechanism, insulin/insulin-like growth factor 1 (IGF-1) signaling, silent information regulator 2 (SIR2), and AMP-activated kinase (AMPK) may act upstream of DAF-16 [42]. To determine if aripiprazole extends lifespan by acting on the insulin/IGF-1 signaling pathway, we first tested the long-lived insulin-like receptor mutant *daf-2*. Aripiprazole failed to extend the lifespan of this mutant (Fig. 6D), suggesting an essential role of DAF-2 in aripiprazole-mediated lifespan extension. The effects of aripiprazole on *age-1*, *akt-1*, and *akt-2* mutants were also examined. However, aripiprazole treatment extended the lifespan of all three mutants by 50.0%, 73.7%, and 50.0%, respectively, which was at a level similar to that of the N2 animals (52.6%) (Fig. 6E–G). These results indicated that aripiprazole did not primarily signal through the insulin/IGF-1 signaling pathway to extend *C. elegans* lifespan. Moreover, aripiprazole increased the lifespan of *sir-2.1* mutant to a similar extent to N2 animals (Fig. 6H), demonstrating that aripiprazole-mediated lifespan extension was also independent of SIR-2.1.

Aripiprazole has been shown to activate mammalian AMPK in PC12 cells [43, 44]. We, therefore, hypothesized that aripiprazole might function through AAK-2 to mediate the extension of lifespan. As shown in Fig. 6I, aripiprazole failed to extend the lifespan of the *aak-2* mutant, suggesting that *aak-2* is required for the effects of aripiprazole on lifespan. We further tested a mutant bearing a mutation in *C. elegans* liver kinase B1 (LKB1) homolog PAR-4, an upstream kinase of AMPK. The *par-4* mutant displayed no lifespan extension upon aripiprazole-treatment (Fig. 6J), which further supported the hypothesis that aripiprazole-mediated lifespan extension requires the activity of AAK-2.

## Discussion

**Fig. 7:**
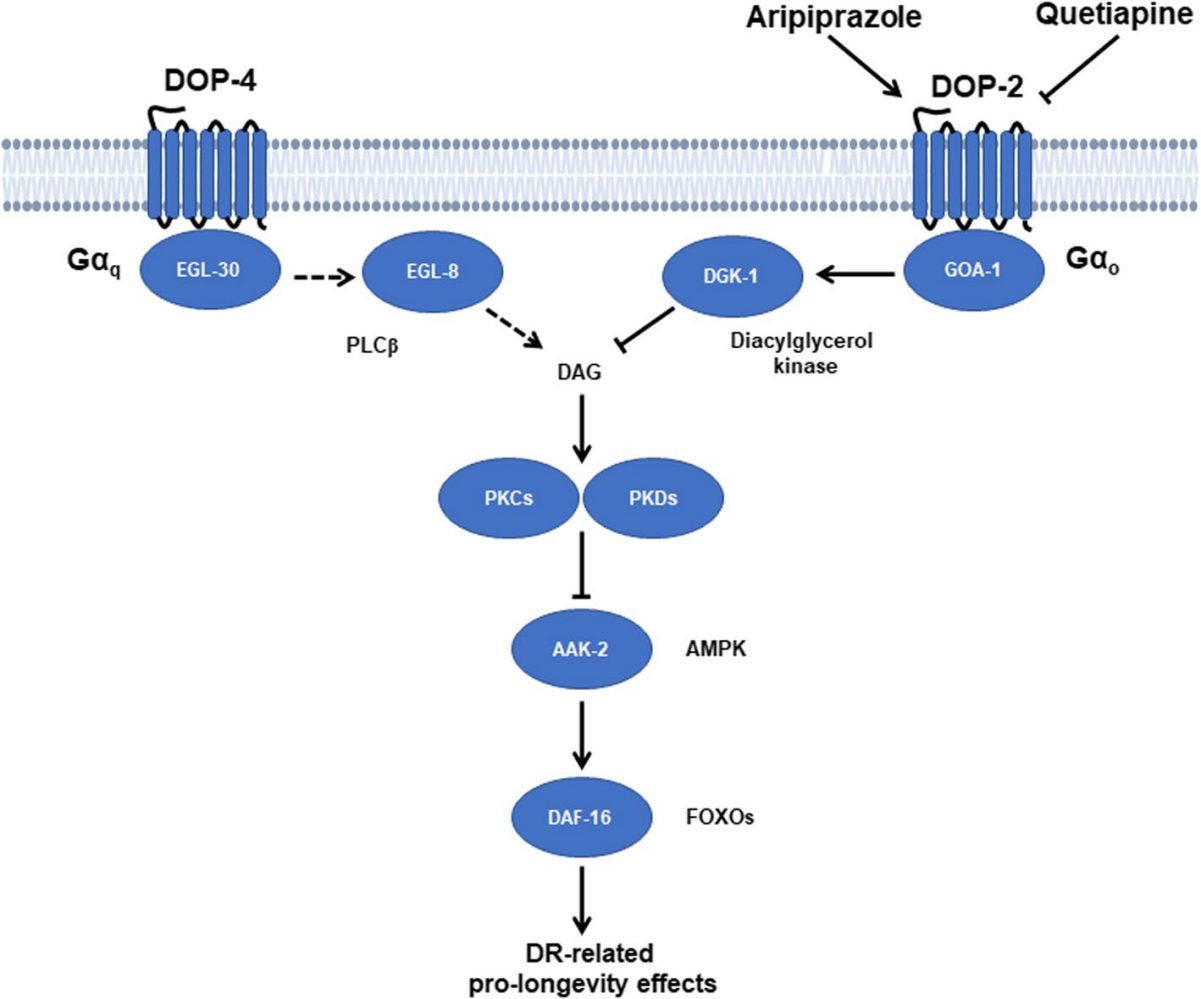
A schematic diagram of the possible mechanism of dopamine receptor signaling-mediated lifespan regulation. In *C. elegans*, DOP-2 signaling extends lifespan while DOP-4 signaling shortens lifespan. Pharmacological activation of DOP-2 using aripiprazole extends lifespan, whereas inhibition of DOP-2 using quetiapine shortens lifespan. Aripiprazole extends lifespan through a DR mechanism mediated by the AAK-2-DAF-16 pathway. The DAG-PKC/PKD pathway served as a link between dopamine receptors and the AAK-2-DAF-16 pathway to transmit longevity signals. The role of DOP-4 in lifespan regulation requires further investigation

In the present study, we showed that D1R (*dop-4*) and D2R (*dop-2*) mutations of *C. elegans* oppositely affected worm lifespan in that the *dop-2* mutant was short-lived, whereas the *dop-4* mutant was long-lived. Pharmacological activation of DOP-2 using aripiprazole robustly extended worm lifespan, whereas inhibition of DOP-2 using quetiapine resulted in shortened lifespan, supporting the selective role of specific dopamine receptors in lifespan regulation. Our data demonstrated that DOP-2 is the major subtype of D2R, and DOP-4 was the major subtype of D1R to regulate lifespan since mutations on the other two dopamine receptors failed to show statistically significant impacts on lifespan. These findings were further supported by the fact that aripiprazole-induced lifespan extension required DOP-2 instead of DOP-3. Mechanistically, dopamine receptors regulate lifespan through a DR mechanism mediated by the AAK-2-DAF-16 pathway. The DAG-PKC/PKD pathway served as a link between dopamine receptors and the AAK-2-DAF-16 pathway to transmit longevity signals. Together, these data represent a novel role of the dopamine system in lifespan regulation, a schematic diagram illustrating the possible mechanism of dopamine system extension of lifespan was shown in Fig. 7.

A previous study reported that several dopamine receptor agonists could extend worm lifespan [45]. The authors found that these compounds did not inhibit the growth of feeding bacterial, indicating that they may extend lifespan by directly acting on the worms [45]. However, the detailed molecular mechanism was unknown. In the present study, we found that aripiprazole extends lifespan by activating the D2R-mediated DR mechanism, indicating that lifespan extensions caused by aripiprazole and possibly other dopamine receptor agonists should be due to their direct effects of modulating worm dopamine signaling; nonetheless, future studies are warranted. In addition, although aripiprazole treatment induced a slight reduction in pharyngeal pumping rate, consistent with a previous report [46], this is also unlikely to be the major cause of DR. First, NP-1, a drug inducing potent lifespan extension through a dietary restriction mechanism, caused a significant reduction in pharyngeal pumping rate in worms but failed to alter food intake [47]. Second, robust lifespan extension usually requires a more significant reduction in pharyngeal pumping rate. The *eat-2(ad1116)* mutant has a 57% longer lifespan than the wild-type animals, similar to that of aripiprazole-treated worms, but featuring a nearly 90% reduction in pharyngeal pumping rate [41, 48]. Two *eat-18* mutants with 70-80% reduction in pharyngeal pumping rate only display 15% and 38% lifespan extension, respectively [41, 48]. Taken together, we provide a novel molecular mechanism explaining how dopamine receptor agonists extend *C. elegans* lifespan. The significant lifespan extension effect caused by D2R activation or loss of D1R signaling should be due to direct activation of intrinsic DR pathways in *C. elegans* instead of altered food intake or inhibited growth of feeding bacterial. Whether D1R agonists and antagonists can affect *C. elegans* lifespan needs further investigation.

As a reasonable extrapolation from the mammalian data that shows the agonistic action of aripiprazole on D2R, aripiprazole should be able to bind DOP-2 in *C. elegans* and stimulate the downstream pathways in the absence of endogenous dopamine. However, interestingly, our results showed that aripiprazole failed to extend the lifespan of the *cat-2* mutant, suggesting that a certain dopamine level may be required for aripiprazole to extend lifespan. A possible explanation is that instead of being a conventional agonist of DOP-2, aripiprazole may act as an allosteric modulator whose binding results in a conformational change of DOP-2 and subsequently enhanced affinity to dopamine. Further studies are needed to clarify the agonistic action of aripiprazole on DOP-2 in *C. elegans*. Notably, our results showed that aripiprazole showed a biphasic effect on *dop-2* mutant that is not presented on other cases. This is also an interesting question that demands further investigation.

The conclusion that aripiprazole extends lifespan through a DR mechanism is supported by the fact that aripiprazole not only failed to extend the lifespan of the *eat-2* mutant but also induced DR phenotypes including prolonged healthspan, reduced brood size, extended reproductive period as well as decreased lipid storage. Further investigation demonstrated that aripiprazole-mediated DR response was dependent on the AAK-2-DAF-16 pathway. DR is subjected to the regulation of multiple genetic pathways, and different DR regimens may elicit different pathways to confer lifespan extension. For example, Greer et al. described that sDR (dilution of feeding bacterial on solid plates) and DP (dilution of peptone) extend lifespan through the AAK-2-DAF-16 pathway, whereas other DR regimens could activate SKN-1, PHA-4 or HSF-1 [49]. In the present study, aripiprazole-mediated DR responses are mechanistically more similar to those induced by sDR or DP. Further studies are needed to identify the precise mechanism of the selectivity of DR on various genetic pathways.

How dopamine signaling is transmitted to the AAK-2-DAF-16 pathway to regulate lifespan is an interesting question. The activity of AMPK can be regulated by a series of upstream kinases, such as PKA, protein kinase B (AKT), PKC, PKD, and LKB1. LKB1 phosphorylates AMPK at Thr-172 for activation. Conversely, PKA, PKC, and PKD phosphorylate AMPK at Ser-485/491 to inhibit its activity [36, 37, 50]. Although we found that LKB1 is required for aripiprazole to extend lifespan, it is unlikely to be directly linked with dopamine receptors. AKT was also excluded since both AKT-1 and AKT-2 were dispensable for aripiprazole-mediated lifespan extension. The cAMP-PKA pathway, a well-known downstream effector of D2R and a target of aripiprazole [51], is also unlikely to play a primary role since ACY-1 and KIN-1 are only partially required for aripiprazole-mediated extension of lifespan. These data indicated that other G protein pathways might be involved.

We found that one possible downstream effector of the dopamine receptors is DAG, which is differentially regulated by Gαq signaling pathway encoded by *egl*-30 and *egl*-8, and the Gαo signaling pathway encoded by *goa*-1 and *dgk*-1. A previous study found that GABA receptor GBB-1 modulates lifespan through the EGL-30-EGL-8-DAF-16 pathway, suggesting a lifespan regulatory role of Gαq signaling pathway [5]. However, the role of Gαo signaling pathway in lifespan regulation has not been reported. Our data demonstrated that aripiprazole-mediated lifespan extension requires all proteins in both Gαq and Gαo signaling pathways. The loss of D1R signaling or activation of D2R signaling may lead to inhibition of worm PKCs and PKDs, and subsequent activation of AAK-2-DAF-16-mediated lifespan extension. Conversely, loss of D2R signaling leads to AAK-2 inhibition and thus shortened lifespan. Our results demonstrated that aripiprazole showed functional selectivity at different *C. elegans* PKC/PKD isoforms. This may be due to the different expression patterns of these isoforms and the tissue-specific effects of aripiprazole.

Dopamine receptors are widely expressed in the nervous system [9]. From mutation studies, it is not feasible to exclude the possibility that alterations of D1R or D2R signaling affect lifespan through a non-cell-autonomous manner. This notion is strengthened by a recent study showing that an olfactory circuit involving dopamine, serotonin, and octopamine signaling mediates dietary restriction by transmitting food odor signals to the gut in *C. elegans* [52]. However, it is known that worm D1R and D2R (DOP-1-4) are not involved in food odor-mediated dietary restriction response. Further investigation is warranted to identify the precise mechanism of D1R and D2R-mediated DR response.

Previous studies based on liquid media have reported that several antidepressants, including mianserin, could extend worm lifespan, mainly by blocking serotonin receptors [53–55]. .A recent work showed that the lifespan-extending effect of mianserin also involves dopaminergic signaling [56]. Despite the antipsychotics, including aripiprazole, have been clinically used for decades, their direct effect on lifespan remains unclear. Some reported that antipsychotics disrupt the development of *C. elegans* [57]. Others reported that antipsychotics, including aripiprazole, could activate the Akt pathway through DAF-2, implying that they may negatively regulate lifespan [58]. Nevertheless, we found that aripiprazole could robustly extend both the lifespan and healthspan of *C. elegans* through a mechanism other than the Akt pathway, but related to dopamine receptor-mediated DR responses. Since DR has been reported to delay the development of *C. elegans* [40], our results may explain the previous finding that treating worms with several other antipsychotics resulted in delayed development, and hinting that starting the treatment from adulthood may be essential for optimizing the pro-longevity of aripiprazole and avoiding its possible developmental toxicity. Notably, aripiprazole has very good long-term safety and tolerability [59, 60]. Moreover, besides its therapeutic effect on psychiatric disorders, aripiprazole also showed a neuroprotective effect, cognitive-enhancing effect, and therapeutic effect against Alzheimer’s disease [61–63]. Therefore, aripiprazole holds potential as a novel safe anti-aging drug.

## Conclusions

Taken together, our findings uncover a novel role of dopamine signaling in lifespan regulation. Genetic inhibition of D1R or pharmacological activation of D2R using aripiprazole could robustly extend both lifespan and healthspan in *C. elegans*. The clinically proved good long-term safety of aripiprazole as well as the fact that the dopamine system and its downstream DR-related pathways is highly conserved among species support the further translation of our finding into humans. Moreover, developing interventions targeting the dopamine system may be a new direction for aging research which aims to benefit human health and longevity.

## Methods

### Nematode *C. elegans* strains and their maintenance

All strains were obtained from the *Caenorhabditis* Genetics Center (CGC, University of Minnesota) and maintained at appropriate temperature on solid nematode growth medium (NGM) plates seeded with *E. coli* OP50. Strains used in this study are described in Additional file 1: Table S1.

### Preparation of reagents

Aripiprazole and quetiapine, purchased from Meilunbio (Dalian, China), were dissolved in dimethyl sulfoxide (DMSO) as stocks. Drugs were added to the liquid NGM before pouring plates. A final DMSO concentration of 0.1% (v/v) was maintained under all conditions.

### Lifespan, fast body movement span, and pharyngeal pumping assays

Worms were cultured for three generations without starvation before lifespan assays. All lifespan assays were performed at 20 °C unless specified. Synchronized late L4 larvae were transferred to lifespan assay plates supplemented with 50 μM of 5-fluoro-2′-deoxyuridine (FUdR, Sigma) to prevent self-fertilization (see Additional file 2: Table S2 for n numbers for each experiment). The day worms were transferred to lifespan assay plates was set as day 0 for an experiment, and worms were counted every 2–3 days. Animals that did not respond to gently prodding by platinum wire were scored as dead. Animals were censored from the experiment if they crawled off the plate or died from vulva bursting or internal hatching (bagging). Lifespan assays were performed in three independent biological replicates. Fast body movement spans were measured along with lifespan assays. Worms with continuous sinusoidal movement when responding to tapping the plates were classified as having fast body movement. For the pharyngeal pumping assay, pharyngeal pumps in the 20s- intervals were recorded under the microscope at day 5 of lifespan assays.

### DAF-16 translocation assay

Strain TJ356 *daf-16(zls356) IV* was used to monitor the translocation of DAF-16:GFP. In each experiment, age-synchronized L4 larvae were treated with 100 μM of aripiprazole or DMSO for 24 h in the same way as described in the lifespan assays. Then, worms were mounted on glass slides with a drop of 0.1% sodium azide and capped with coverslips. Images of the DAF-16:GFP signal was quickly taken with a Nikon TiE fluorescent microscope. Animals were scored as having cytosolic or nuclear localization when localization was observed throughout the entire body or intermediate localization when a mixed distribution pattern was shown. This assay was repeated at least three times and scored by two different individuals.

### Brood size and reproductive lifespan assays

Worms (*n* = 10 or 15) were transferred to fresh NGM plates with bacterial lawn. Each worm was placed in one plate and transferred to a fresh NGM plate every 24 h until egg-laying had ceased. The number of hatched worms on each day was counted after 72 h of incubation at 20 °C. The brood size of each worm was the total number of hatched progenies during the assay. The reproductive lifespan of each worm was the time period that it was capable of laying eggs.

### Developmental rate assay

Synchronized eggs of each strain were placed on NGM plates and cultured at 20 °C. Developmental stages of animals were visually inspected under a stereoscope after 2.5 days. 50 to 100 animals were scored for each strain.

### Oil red O (ORO) staining

About 1000 age-synchronized L4 larvae were transferred to 6 NGM plates containing 100 μM of aripiprazole or DMSO and cultured for 7 days at 20 °C. Worms were collected, washed with phosphate buffered saline (PBS), and fixed in 2 × MRWB-PFA. Then, worms were washed and dehydrated in isopropanol. After that, isopropanol was replaced with a new ORO solution. After ORO staining, animals were mounted on slides, and images were taken by a Carl Zeiss Axio Imager 2.

### Statistical analysis

The lifespan assays were analyzed using the Kaplan-Meier method and the log-rank test. Brood size assay, pharyngeal pumping rate assay, and ORO staining were analyzed using unpaired *t*-tests. Error bars were presented as mean ± SEM. Statistical analyses were performed using GraphPad Prism 8 (GraphPad Software, San Diego, CA).

## Supplementary Information

**Additional file 1: Table S1.** List of *C. elegans* strains used in this study.

**Additional file 2: Table S2.** Supporting data.

## Data Availability

All data generated or analyzed during this study are included in this published article and its supplementary information files. Supporting data are included in Additional file 2.

## Abbreviations

*AKT*: Protein kinase B
*AMPK*: AMP-activated protein kinase
*Ari*: Aripiprazole
*cAMP*: Cyclic AMP
*C. elegans*: *Caenorhabditis elegans*
*DAG*: Diacylglycerol
*D1R*: Dopamine D1-like receptor
*D2R*: Dopamine D2-like receptor
*DMSO*: Dimethyl sulfoxide
*DR*: Dietary restriction
*FUdR*: 5-Fluoro-2′-deoxyuridine
*GABA*: Gamma-aminobutyric acid
*GFP*: Green fluorescent protein
*IGF-1*: Insulin-like growth factor 1
*NGM*: Nematode growth medium
*LKB1*: Liver kinase B1
*ORO*: Oil red O
*PKA*: Protein kinase A
*PKC*: Protein kinase C
*PKD*: Protein kinase D
*Que*: Quetiapine
*SIR2*: Silent information regulator 2
